# Improving the efficacy of selenium fertilizers for wheat biofortification

**DOI:** 10.1038/s41598-019-55914-0

**Published:** 2019-12-20

**Authors:** Chandnee Ramkissoon, Fien Degryse, Rodrigo C. da Silva, Roslyn Baird, Scott D. Young, Elizabeth H. Bailey, Mike J. McLaughlin

**Affiliations:** 1 Fertiliser Technology Research Centre, School of Agriculture, Food and Wine, Waite Campus, University of Adelaide, Adelaide, South Australia Australia; 2 School of Biosciences, University of Nottingham, Sutton Bonington Campus, Loughborough, UK

**Keywords:** Environmental social sciences, Biogeochemistry

## Abstract

Increasing the selenium (Se) concentration of staple crops by fertilization is a valuable pathway to increase Se in the human diet, thus preventing Se deficiency. A pot trial was set up to investigate whether the application of 3.33 µg kg^−1^ of Se (equivalent to 10 g ha^−1^) to wheat can be made more efficient by its co-application with macronutrient carriers, either to the soil or to the leaves. In the soil, Se was applied either on its own (selenate only) or as a granular, Se-enriched macronutrient fertilizer supplying nitrogen, phosphorus, potassium or sulfur. Selenium was also applied to leaves at head emergence with, or without, 2% w/v N fertilizers. With grain Se concentrations varying from 0.13–0.84 mg kg^−1^, soil application of selenate-only was 2–15 times more effective than granular Se-enriched macronutrient fertilizers in raising grain Se concentrations. Co-application of foliar Se with an N carrier doubled the Se concentration in wheat grains compared to the application of foliar Se on its own, the majority of which was in the highly bioavailable selenomethionine fraction. Results from this study demonstrate the possibility of improving the efficacy of Se fertilizers, which could enrich crops with Se without additional application costs in the field.

## Introduction

The essentiality of selenium (Se) as a nutrient for humans and animals was first established in the 1950s by Schwarz and Foltz^1^. Since then, its active role as an antioxidant, thyroid hormone and general immune function regulator has been highlighted, such that a low intake of Se in the diet would result in poor health and in extreme cases, deficiency diseases^2^. Although less common, an excess of Se can also be detrimental to human health^3^. There is a narrow margin between Se deficiency and toxicity and so it is essential that the daily dietary Se intake for humans falls within a restricted range. Currently, the recommended dietary intake is 50–55 µg day^1–4^, but it is estimated that 0.5–1 billion people around the world do not consume sufficient Se and are at risk of disease^5,6^.

Agronomic biofortification is the practice of increasing the nutrient concentration of the edible parts of staple crops through fertilization practices^7^. In recent decades it has been identified as an effective long-term strategy to alleviate micronutrient deficiency because it is relatively easy, efficient and affordable^8^. Cereals, such as wheat and rice, are ideal for Se biofortification because they are widely consumed by the general population and they can act as effective buffers for humans since they accumulate no more than 1.0 mg Se kg^−1^ of dry matter^9^.

The form in which Se is applied affects its effectiveness for biofortification. Both selenate (Se^VI^) and selenite (Se^IV^) are bioavailable species but the uptake rate of Se^VI^ may be up to 33 times higher than that of Se^IV^ ^10^. This is because Se^IV^ is adsorbed more strongly by inner-sphere complexation onto soil mineral oxides/hydroxides surfaces, which limits its mobility and hence plant uptake^11^. Moreover, Se^IV^ has limited translocation through plants and tends to accumulate in roots, compared to Se^VI^ which is highly mobile in the xylem^12^. The predominance of the different species in soils in turn depends on *in-situ* factors such as the soil geocolloidal phases present, pH and redox potential. Under high pH and well aerated conditions, such as arable soils, Se^VI^ is expected to be the dominant inorganic Se species while in more acidic well-drained soils or under anaerobic conditions, Se^IV^ concentrations are expected to be greater^13^.

Selenium fertilizers are typically applied at low rates of 10–20 g Se ha^−1^ in biofortification studies^14^. To ease the application of such a small amount of Se in the field, it is usually added to other fertilizer matrices, supplying either a mix of nutrients, for example Selcote Ultra and Top Stock^7^, or predominantly macronutrients, such as urea and calcium nitrate^15^. These fertilizer matrices are referred to as “carriers” of Se. In 1993, Gupta *et al*.^16^ investigated the application of nitrogen (N) fertilizers ammonium nitrate (NH_4_NO_3_) and urea doped with either Se^IV^ and Se^VI^ to improve the Se levels of livestock. While their main findings focused on the superiority of Se^VI^ compared to Se^IV^ in increasing plant Se levels, they also pointed out that both N fertilizers were effective as carriers for Se. Additionally, Premarathna *et al*.^8^ reported that Se-enriched urea granules were very effective in raising Se concentration of rice, hence highlighting the potential of N as a carrier for Se. Rice however has different growth conditions to cereals crops such as wheat, such that findings from such an experiment may or may not be transposed onto other crops. To our knowledge, no study had either investigated this carrier effect with wheat or compared the efficiency of different macronutrients as Se carriers.

A few studies have compared the efficiency of applying Se by different methods – to the soil or to the leaves (foliar). Results showed that, while both are effective in raising plant Se concentrations, foliar fertilization is up to 8 times more efficient than soil Se application^10^. This greater efficiency of foliar-applied fertilizers may be ascribed to (1) rapid uptake and assimilation due to application at a later growth stage, (2) less influence of root-to-shoot ratio on translocation to the edible parts of crops and (3) the avoidance of losses through fixation in soils. On average, only 12% of soil-applied Se fertilizers is taken up by plants; most Se applied is retained and immobilized in the soil^7^, with very little residual value for subsequent crops^17^. This means that repeated applications of Se fertilizers are required for each growth period, unless the efficacy of Se fertilizers can be improved.

In this study, we investigated the potential for enriching commonly used fertilizers supplying macronutrients nitrogen, phosphorus (P), potassium (K) and sulfur (S), with Se to biofortify crops. We hypothesized that macronutrients can act as effective carriers for Se and help improve fertilizer use efficiency in the field. We believe this is the first study investigating the efficiency of N, P, K and S as well as water as carriers for Se, applied either to the soil or to the leaves, with the aim of increasing Se levels in wheat grains. In addition, we did Se speciation analysis of the wheat grains to determine whether the different fertilizer formulations had an effect on the bioavailable Se content of the wheat grain.

## Results

### Macronutrient concentration

Despite the application of macronutrient fertilizers in different ways (either as granules or as a basal solution) in this experiment, all treatments received the same rate of macronutrient N, P, K and S application. Hence, no significant differences were observed in the macronutrient content of the grain, except for the granular DAP-Se treatment in the KI soil, which showed a higher grain P concentration (3.51 ± 0.17 g kg^−1^) than when P was applied in the basal solution (2.70 ± 0.07 g kg^−1^) (Supplementary Fig. S1). Slight, although statistically significant, differences in grain K concentration were observed between some treatments in KI and Mallala soils, whereby foliar treatments seemed generally higher than soil-applied ones. However, in all these treatments, a similar rate and method of K fertilizer (MOP in basal solution) was applied; any differences observed were therefore attributed to random effects.

### Yield and Se concentration

**Figure 1:**
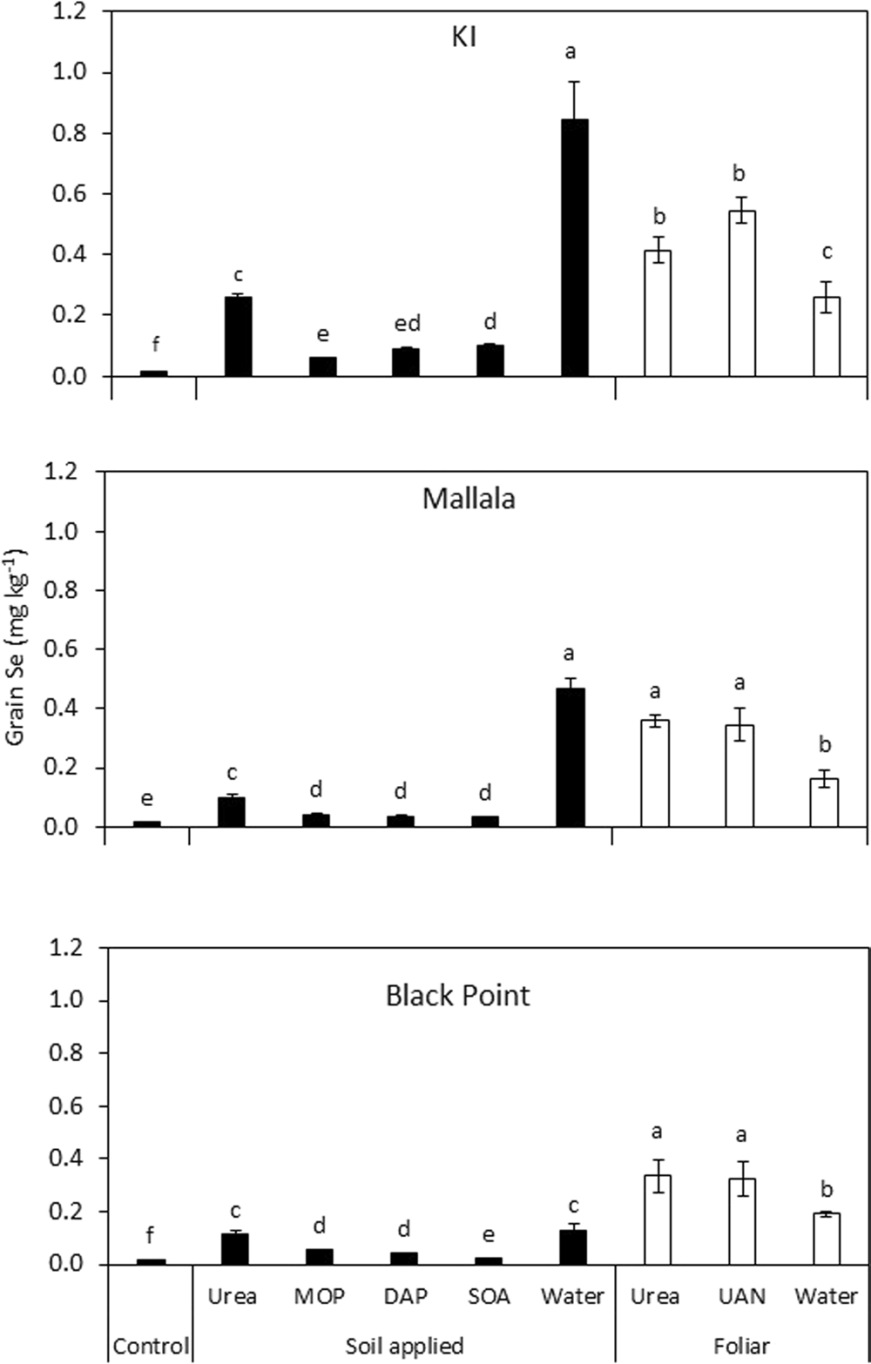
Grain Se concentration across different Se fertilization treatments used in the three soils. Under soil-application, Se was applied with N, K, P and S fertilizer carriers as Se-enriched urea, MOP, DAP and SOA granules respectively. Also a treatment with water as carrier (spot-applied sodium selenate solution) was included. Results show means and standard errors (n = 4). Different letters above the bars indicate significant (p ≤ 0.05) differences between treatments (Duncan multiple range test) at a 5% significance level.

Irrespective of their formulation and method of application, the different Se fertilizers employed in the study did not significantly affect grain yield, which ranged from 3.5–4.2 g pot^−1^ for the three soils (Supplementary Fig. S2), but significantly increased grain Se concentrations above control levels (Fig. 1). A similar pattern in Se accumulation across the treatments was observed in the three soils, although plants grown in the KI soil generally had higher Se concentrations than Mallala- and Black Point-grown ones. For the soil-applied treatments, the application of Se on its own was the most effective (0.84 ± 0.01 mg kg^−1^ in KI; 0.46 ± 0.04 mg kg^−1^ in Mallala and 0.13 ± 0.02 mg kg^−1^ in Black Point) followed by granular Se + urea treatments (0.26 ± 0.11 mg kg^−1^ in KI; 0.10 ± 0.01 mg kg^−1^ in Mallala and 0.11 ± 0.02 mg kg^−1^ in Black Point) (Fig. 1). In comparison, soil application of Se with the other macronutrients P, K and S had a much smaller effect on Se accumulation in the plants. Grain accumulation of Se following foliar fertilization was consistently higher when 2% w/v N, in the form of urea or UAN, was added to the foliar Se solutions (Fig. 1): grain Se concentrations under the foliar Se only treatment averaged at 0.20 ± 0.02 mg kg^−1^, which compared to 0.37 ± 0.02 mg kg^−1^ and 0.41 ± 0.07 mg kg^−1^ when foliar Se was co-applied with urea and UAN, respectively. The use of either liquid urea or UAN were equally effective in enhancing grain Se accumulation. No Se was measured in the foliar rinses of the treated leaves, suggesting that the surface-applied Se had been absorbed into the leaves.

### Nitrogen content and speciation

**Figure 2:**
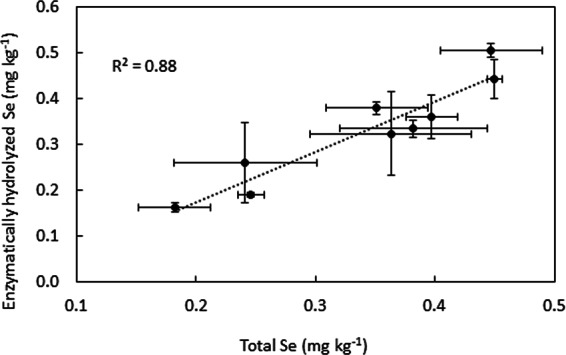
Correlation between total grain Se concentrations measured by two methods: acid digestion and enzymatic hydrolysis. Error bars represent standard errors (n = 4).

Grain N was around 2.1% of the total weight across the different treatments where N was analyzed, except when Se-enriched urea granules were soil-applied in KI soil, which resulted in higher grain N content (3.53%) (Supplementary Fig. S3). Protease hydrolysis of the grains measured 104 ± 4.39% of the total Se, suggesting that it was a reliable way of releasing Se from the grains (Fig. 2), the majority of which was in SeMet form (average 97 ± 6%). The distribution of SeMet therefore followed that of the total Se (Supplementary Fig. S4), suggesting that the use of different carriers and methods of application did not affect speciation of Se in the grains. Other Se species such as selenocysteine (SeCys) and Se-methyl-selenocysteine (MeSeCys) generally found in wheat grains were not quantified in this study, but it is likely that that the small percentage of unidentified Se species in the grains was in organic form^18^.

### Selenium recovery and translocation to grains

**Figure 3:**
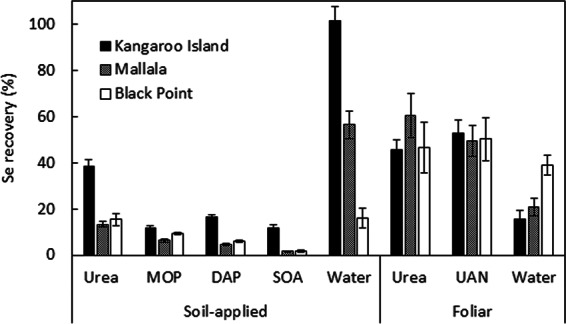
Percentage of applied Se fertilizer recovered in aboveground biomass. Error bars show standard errors (n = 4).

Generally, the recovery of fertilizer in the aboveground biomass was less than 50% when Se fertilizers were applied to the soil, except for soil-applied selenate-only in KI and Mallala soils (100% and 56% respectively; Fig. 3). Although the roots or the soils were not analyzed for Se concentrations in this study, we believe that the rest of the applied Se might either be stored in the roots or lost to the environment either through a retention mechanism onto soil particles or volatilization from the plants^7,19^. Crop Se recovery was especially low (2–38%) when Se was applied to the soil with macronutrient fertilizers, with the highest recovery recorded for the soil-applied Se + urea treatment in KI. The foliar Se fertilizers were more efficient in accumulating Se in crops with 19–30% and 46–61% Se recovered in the harvested biomass when Se was applied on its own and with an N carrier, respectively.

**Figure 4:**
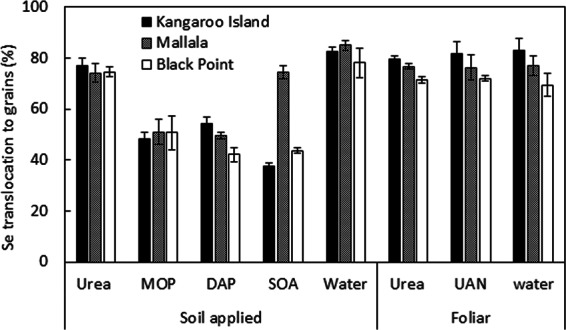
Percentage of Se translocated to the grain across the different fertilizer treatments used in the three soils. Error bars show standard errors (n = 4).

To examine translocation of Se into grain, the uptake (Se concentration x grain dry weight) of Se by wheat grains was expressed as a percentage of the total amount of Se accumulated in the aboveground biomass (grains + shoots). Our results showed that when Se fertilizer was soil-applied with water or with an N carrier, >75% of the Se fertilizer taken up in the aboveground biomass was translocated to the grains (Fig. 4). On the other hand, limited translocation ( <50%) was observed when Se was applied with MOP, DAP and SOA (except in Mallala). The foliar applications, both with and without N, showed a large translocation to the grain.

## Discussion

Yield did not differ significantly across treatments in this study, in agreement with previous studies when rates of up to 100 g ha^−1^ of Se have been applied^7,20,21^ (Supplementary Fig. S2). In other, albeit fewer, instances where a positive relationship between Se application and plant yield was observed, the response was attributed to a stimulation of antioxidant activity and subsequent plant protection from abiotic stresses such as cold, desiccation and the presence of toxic metals^22^. The essentiality of Se for higher plants is still unconfirmed; it is generally thought to be beneficial for several physiological processes but is not a limiting factor for growth^23^.

**Figure 5:**
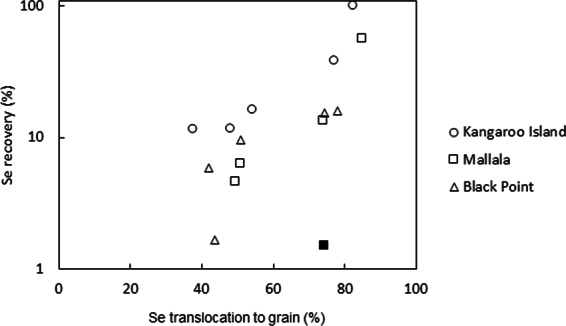
Percentage of Se recovered in the aboveground biomass vs. % of Se translocated to the grain of plants fertilized with soil-applied Se-enriched macronutrient fertilizers (urea, MOP, DAP and SOA). The single filled data point indicates the Se-enriched SOA treatment in Mallala soil.

Grain Se concentration of control plants in this study was very low, averaging 0.015 ± 0.00 mg Se kg^−1^, which is below the target Se concentration of 0.1 mg kg^−1^,suggested to be adequate for human consumption^24^ (Fig. 1). Under soil application treatments, the effectiveness of the Se fertilizers depended on the macronutrient carrier as well as the soil characteristics. When Se was co-applied with macronutrient fertilizers such as MOP, DAP and SOA as granules to the soil, most (>90%) of it remained unutilized by the crop. Recovery rates of Se in those soil-applied treatments were lower than the average 12–27% reported by Stroud *et al*.^25^ and Broadley *et al*.^7^ but compared favorably with rates in the field trial by Stephen *et al*.^26^ who reported 6.9% to 4.9% recovery in autumn-grown wheat (Fig. 3). However, unlike their autumn field trial, where considerable amounts of the applied Se fertilizer might have been lost by leaching^26^, ours was a pot trial conducted under controlled conditions. This suggests that mechanisms other than leaching, for example, sorption by soil, were responsible for the poor efficiency of Se-enriched macronutrient fertilizers. The exact mechanism explaining their poor efficiency compared to the application of selenate on its own to the soil is not known yet, but a possible explanation might be that the reduction of Se^VI^ to Se^IV^ was faster for the granular treatments. Since Se^IV^ is more strongly sorbed to soil hydrous oxides and organic matter and has a relatively low root-to-shoot translocation compared to Se^VI^ ^27,28^, its predominance in the soil would explain the low Se uptake in the plants. A positive relationship between Se translocation and Se recovery was observed (Fig. 5), which supports this hypothesis. The low Se translocation for the treatments with low recovery (with the exception of Se-enriched SOA in Mallala soil) suggests that Se^IV^ was the predominant species available for plant uptake in these treatments. This change in Se chemical speciation could have been because, as the fertilizer granule dissolved in the soil and salt concentration built up, water would flow towards the granule as a result of the high osmotic pressure^29^, and that could create a locally reducing environment. For Se-enriched urea granules, this mechanism might be less relevant because urea is initially uncharged and even though its hydrolysis is rapid^30^, the urea would already have started to diffuse away from the application site before hydrolysis, resulting in less osmotically-driven water flow towards the application site. Moreover, the consumption of H^+^ ions during urea hydrolysis (NO_3_^-^ assimilation) is usually accompanied by a temporary increase in soil pH^31^. All these conditions would tend to favor the predominance of Se^VI^ ions, which could explain the higher Se uptake when urea was co-applied with Se compared to the other macronutrient fertilizers.

For the treatment with Se-enriched SOA granules in the Mallala soil, a very low Se recovery (2%) was recorded in the aboveground biomass of these plants despite the high translocation of Se to the grain (Fig. 5). While the high translocation rate suggests that Se^VI^ was the predominant species available for uptake, probably because roots were exposed to alkaline aerobic conditions^13,32^, the low Se recovery suggests that the uptake of Se from the soil was restricted. The negative effect of S fertilizer on grain Se uptake has been documented before^33^; the antagonism arises as a result of the competition between chemically similar selenate and sulfate ions for uptake transporters in the root, where sulfate is preferentially taken up to selenate due to its higher affinity for the transporters^32,34^. More recent studies by Tan *et al*.^35^, investigating novel mechanisms behind the competitive relationship between sulfate and selenate showed that the reduced plant uptake of selenate in the presence of sulfate ions could also be due to a suppression in microbial ability to assimilate Se^VI^. In our study, even though sulfate and selenate were applied at the same rate for all treatments, their close proximity in Se-enriched SOA granules potentially enhanced the competition, thus reducing the uptake of Se.

In comparison to the application of Se with macronutrient carriers, the application of Se^VI^ on its own to the soil was far more effective in increasing grain Se concentration (high Se recovery and high translocation to grain), especially in the KI and Mallala soils. We suggest three possible explanations for this phenomenon: (1) there was potentially a lower propensity for Se^VI^ to be reduced to Se^IV^ as a result of the lower osmotic pressure (no granule dissolving); (2) there was a lack of competition between ions since Se^VI^ was applied in pure form and; (3) there was no added physical restriction of Se having to diffuse out of the granule when it was applied in pure fluid form to the soil. Despite the granular fertilizers being highly soluble in water (Table 1), the dissolution of the individual granule in the soil might have been slower than expected, hence restricting Se release.

**Table 1:** Water solubility of Se-enriched macronutrient fertilizers.

Se-enriched fertilizer	Water-soluble Se mg kg^−1^	Acid-soluble Se mg kg^−1^	^a^Water solubility %
Urea	34.7	32.3	107 ± 0.92
MOP	29.3	31.6	93 ± 0.36
DAP	25.8	26.7	96 ± 0.24
SOA	37.7	36.3	104 ± 0.25

^a^Water solubility is presented as a percentage of the total Se released by acid digestion.

Under soil-applied Se treatments, plants grown in KI soil accumulated more Se compared to those grown in Black Point or Mallala soils (Fig. 1), indicating that soil properties affected the effectiveness of the fertilizers. Soil properties can affect mobility and availability of Se for plant uptake through their effect on soil conditions (e.g. pH and pe), which in turn affects Se chemical speciation and sorption behavior. Under high soil pH and aerobic conditions, Se^VI^ ions would predominate in the soil, which would favor plant uptake because Se^VI^ is adsorbed to a much lesser extent on geocolloids compared to Se^IV^, which makes it more mobile and bioavailable^36^. However, in soils with such conditions (good aeration and high pH) for example Mallala, Se uptake was lower than expected, suggesting that other factors, such as CaCO_3_, might have limited Se bioavailability. Previous studies have shown that Se^IV^ can get adsorbed onto calcite surfaces via an anion exchange mechanism as CO_3_^2−^ and SeO_3_^2−^ have a similar charge and ionic radius^37^. Soil texture and organic matter content are also factors which can influence Se bioavailability. With only 5% clay content, KI soil is very sandy (Table 2), which, not only makes it more likely to be well aerated, hence promoting the predominance of mobile Se^VI^ ions, but also lowers its adsorption capacity, compared to the Black Point and Mallala soils.

**Table 2:** Physicochemical properties of the three soils used in this pot experiment.

Soils	^a^EC dS m^−1^	pH	CaCO_3_ %	Clay %	Sand %	Organic C %	Exchangeable cations (cmol_c_ kg^−1^)				
							^b^ECEC	Ca	Mg	Na	K
Kangaroo Island	0.07	5.5	<0.5	5	94	1.6	2.71	2.09	0.62	<0.1	<0.2
Mallala	0.13	8.5	4.7	11	47	1.6	30.8	26.2	2.58	0.16	1.85
Black Point	0.07	8.3	<0.2	18	73	0.4	17.9	14.2	2.54	0.17	0.97

^a^Electrical conductivity (EC) of soils.

^b^Effective cation exchange capacity (ECEC) of soils.

The foliar application of Se fertilizers tended to be more efficient than the soil application, with higher Se uptake and recovery rate in the plants (Fig. 3). In this study, a foliar application equivalent to 10 g Se ha^−1^ led to grain concentrations of 0.1–0.3 mg kg^−1^ when Se was applied on its own and up to 0.5 mg kg^−1^ when Se was applied with an N carrier to the leaves (Fig. 1). These concentrations compare favorably with the average Se concentration of 0.4–0.5 mg kg^−1^ measured in studies by Curtin *et al*.^38^ and Ducsay *et al*.^39^, where twice the amount of Se (20 g ha^−1^) was applied to the leaves. Thus there is clearly greater efficiency in co-applying foliar Se with an N carrier to enrich wheat grain with Se, although the reasons for this have not yet been established. In studies looking at the effect of co-applying trace elements such as Fe and Zn with N, the N nutritional status of the plants was given as an explanation for improved grain micronutrient uptake because proteins can act as a sink for micronutrients and aid their re-translocation from shoots to the grain^40,41^. However, our study showed that the addition of 2% w/v N in foliar solutions did not significantly alter grain N (protein) content (Supplementary Fig. S3), suggesting that a physiological mechanism may instead be responsible for the improved plant uptake when foliar Se was co-applied with N. The physiological response might have improved Se absorption into the leaf and/or improved translocation into the grains. Nitrogen fertilizers such as urea and UAN are often foliar-applied as they are uncharged molecules which can easily permeate waxy leaf cuticle though a simple diffusion mechanism^42^. Co-applying Se with such N carriers potentially facilitated the Se sorption pathway. Moreover, once absorbed, N and Se have a similar assimilation pathway in plants in the sense that both get metabolized into N organic compounds such as amino acids. Therefore, co-applying Se with a N carrier potentially improved its rate of assimilation into selenoamino acids, which would then be transported into sink organs (grains). Comparatively, when applied without a N carrier, Se may take a longer time to penetrate the cuticular membrane and get assimilated, leaving a greater window of opportunity for losses by (phyto)volatilization^43^. Effectively, losses of Se under foliar Se-only treatment were twice as much as those under foliar Se + urea and Se + UAN treatments (Fig. 3). To the best of our knowledge, this is the first study showing an improved plant Se uptake when Se was foliar-applied with a N source. Similar effects have been observed with other micronutrients, for example, in studies by Aciksoz *et al*.^44^, where improved Fe translocation from the foliar-treated leaf to the grain was observed when Fe was co-applied with up to 0.8% w/v urea to wheat plants.

## Conclusions

Our study aimed to determine whether fertilization strategies for Se biofortification could be made more cost-effective by co-applying Se with commonly used macronutrient fertilizers. It was observed that the effectiveness of those Se-enriched fertilizers was highly dependent on soil properties and that the co-application of Se with macronutrients in granular form generally led to poor Se uptake and translocation within the plant. In two of the three soils used in this experiment, the application of selenate on its own to the soil was more effective in increasing grain Se concentrations than any other soil-applied fertilizer strategy. Our study also showed that foliar application of Se with 2% w/v N can lead to twice as much Se uptake and recovery in plants, compared to foliar application of Se only. It should be noted that foliar solutions were applied as targeted droplets on specific leaves in this pot trial, and that, in the field where foliar sprays would be used, lower Se recovery rates can be expected. However, it appears likely that foliar co-application of Se with a N carrier would still be more effective in raising grain Se concentrations compared to foliar Se only or soil-applied Se-enriched macronutrient fertilizers.

## Materials and Methods

### Soils

The experiment used three Australian soils, Kangaroo Island (KI), Mallala and Black Point, air-dried and sieved to < 2 mm. They were chosen to provide a range of physical and chemical characteristics likely to affect Se dynamics (Table 2). Soil pH and electrical conductivity (EC) were measured in a 1:5 soil-to-solution suspension on an automated Skalar pH/EC system. Soil organic carbon (C) content was measured using a dry combustion method^45^. The textural classification of the soils were determined using mid-infrared spectroscopy and R code to generate the classification from the Australian soil textural triangle. To determine the exchangeable cations contents and effective cation exchange capacity (ECEC), the soil samples were shaken with a 1 M ammonium acetate solution at pH 7 in a 1:10 soil-to-solution ratio and the extracts were analyzed for elemental concentrations using inductively coupled plasma optical emission spectrometry (ICP-OES) (Optima 8300; PerkinElmer Inc., Waltham, Massachusetts).

### Selenium fertilizers

Based on application suggestions from previous biofortification studies^38^, Se was applied as sodium selenate (Na_2_SeO_4_) at a single rate of 3.33 µg Se kg^−1^ (equivalent to 10 g ha^−1^, based on a 20 cm depth and 1.5 g cm^−3^ bulk density). There were nine treatments for each soil, each replicated four times. Treatments included: (i) a control without added Se, (ii) a treatment with Se added to soil as sodium selenate solution, (iii) four treatments with Se-enriched granular fertilizers and (iv) three treatments with foliar Se fertilizer.

The granular fertilizers used were urea, di-ammonium phosphate (DAP), muriate of potash (MOP) and sulfate of ammonia (SOA), supplying the macronutrients N, P, K and S respectively. To enrich these fertilizers with Se, a sodium selenate solution was added to powdered commercial fertilizer and mixed thoroughly to ensure homogeneity. The paste was then oven-dried overnight at 30 °C and ground to a fine homogenous powder using a pestle and mortar. The Se-enriched fertilizer powder was then pressed into tablets (5 mm diameter, ca. 2 mm height) using a tablet press (TDP-5, Shanghai Develop Machinery Co., China). For the treatment with the soil-applied selenate only, a Na_2_SeO_4_ solution containing 0.042 mg Se L^−1^ was applied to the soil as 3 × 26 µL droplets, in the same position as the granular fertilizers.

Foliar treatments included a Se-only solution (water as carrier), Se + N in the form of either 2% w/v urea or 2% v/v urea ammonium nitrate (UAN). All three solutions contained Se as sodium selenate at a concentration of 0.083 g Se L^−1^ (rate equivalent to 3.33 µg Se kg^−1^) and were mixed with 0.5% “Spreadwet 1000” (SST Australia PTY LTD., Victoria, Australia) surfactant prior to application.

### Pot trial

All soils were mixed with the following nutrients (mg kg^−1^ of soil): Ca (10), Mg (10), B (1.0), Cu (2.0), Mn (2.0), Mo (0.1) and Zn (2.0) and left to equilibrate overnight prior to potting into 1 kg pots. Macronutrients were also supplied, including 80 mg kg^−1^ N as a split application, 20 mg kg^−1^ P and S, and 40 mg kg^−1^ K. The application method of the macronutrients depended on the treatment; when enriched with Se, the macronutrient fertilizer was applied as granules (3-4 per pot) in a circle at a distance of 1 cm from the side of the pot halfway through potting. The other macronutrients were then applied as part of the basal solution, such that, regardless of their form of application, all nutrients were balanced in all the soil pots. After fertilization, five pre-germinated wheat seedlings (*Triticum aestivum cv*. Axe) were transplanted into each pot and thinned to two plants after two weeks. The soils were maintained close to field capacity by watering the soil surface regularly with reverse osmosis (RO) water. At heading stage, foliar solutions were applied to the youngest flag leaf as four 5-µL drops per plant using a micropipette. The soil surface was covered with cling film to avoid any contamination during foliar application and care was taken to water the plants at the soil surface only, avoiding irrigation of leaves. Plants were grown to grain maturity under controlled conditions (temperature of 23.2 °C, humidity of 72% and 12 h daylight cycle).

### Harvest

At grain maturity, shoots and heads were harvested separately. Marked treated leaves were also separated from the rest of the biomass and washed in dilute hydrochloric acid (HCl; 0.1 M) and then rinsed with reverse osmosis (RO) water; acid rinses were saved and analyzed for Se. All plant biomass was dried at 60 °C for 72 h, after which wheat heads were hand-threshed to separate grains. Prior to analyses, the grains were ground to fine powder using a pestle and mortar, and the rest of the head biomass was combined with the shoots and ground using a laboratory grade grinder.

### Analyses

#### Fertilizers

Total Se concentration in the fertilizers was measured following acid digestion. Two mL of concentrated nitric acid (HNO_3_) and 0.5 mL of 30% hydrogen peroxide (H_2_O_2_) was added to 0.25 g of Se-enriched fertilizer and left to stand overnight. The samples were then heated to 80 °C for 45 min followed by 125 °C for 160 min on a block digester. After acid digestion, the samples were cooled for 30 min then made to 10 mL volume using ultrapure Milli-Q water. To measure water-soluble Se in the fertilizer, 0.5 g of granular Se-enriched fertilizer samples was dissolved in 10 mL of Milli-Q water and the mixture was shaken end-over-end for 4 h. The samples were then centrifuged (15 min at 3000 *g*) and filtered through 0.22 µm filters (Sartorius, Göttingen, Germany). All solutions were analyzed for total Se by ICP-OES.

The water solubility test of our Se-enriched fertilizers indicated that they were highly soluble, releasing 100 ± 10% of the added Se in water (Table 1).

#### Plants

Approximately 0.25 g of plant sample (4 replicates) were weighed into 50 mL digestion tubes (Axygen, Thermo Fisher Scientific, New York) and left overnight in 2 mL of HNO_3_ acid and 0.5 mL of H_2_O_2_ to predigest. The samples were digested using the same method as for the fertilizers, cooled and made to a final volume of 20 mL with Milli-Q water.

The acid digests were analyzed after hydride generation using a Multimode Sample Introduction System (MSIS) (Agilent Technologies, Victoria, Australia) mounted onto conventional ICP-OES^46^. Since only selenite forms hydrides, all samples were pre-reduced to Se^IV^ by heating an aliquot (5 mL) of the acid digest with an equal volume of concentrated HCl at 90 °C for 30 min prior to analysis. Other elements (Ca, Cu, Fe, K, Mg, Mn, P, S, and Zn) were analyzed by conventional ICP-OES, after a 5-fold dilution of the plant acid digests.

Analytical accuracy was verified through the analysis of wheat flour certified reference materials, NIST 8437 and NIST 1567b (National Institute of Standards and Technology, Maryland). The total Se concentration of the reference materials was within the range 90–110% recovery of the certified values.

After initial analysis, grain samples with the highest measured Se concentration (from foliar and soil-applied selenate-only treatments) were analyzed for total N content and Se speciation. Grain nitrogen was determined by the combustion (Dumas) method, as described by Horneck and Miller^47^, and analyzed on an N analyzer (Model Leco FP-528L 601-500-100; Leco Corporation, St Joseph, Michigan). For Se speciation, 0.2 g of ground grain was weighed into 15 mL polypropylene tubes with 20 mg of protease XIV enzyme (Sigma-Aldrich, Queensland, Australia) and dissolved in 5 mL of 30 mM TRIS-HCl buffer solution. The solution pH was adjusted to 5.5 using ammonia (NH_3_) solution. The samples were shaken end-over-end in an incubator at 37 °C for 24 h, centrifuged at 3000 *g* for 30 min and filtered through 0.22 µm filters. The resulting solutions were analyzed for Se^IV^, Se^VI^ and SeMet using high-performance liquid chromatography coupled with inductively coupled plasma mass spectrometry (HPLC-ICPMS, Agilent 7500ce, Agilent Technologies). The operating conditions were adapted from Premarathna *et al*.^8^ (Supplementary Table S1). The concentration of Se species in the samples was determined by comparison of their retention times with those of standards, prepared from individual and mixed stock solutions of sodium selenite (Na_2_SeO_3_), Na_2_SeO_4_ and selenomethionine (SeMet).

Recovery of the applied Se in the plants (Se_recovery_; µg pot^−1^) was calculated as the total amount of Se measured in the aboveground biomass as a percentage of the applied Se fertilizer (Eq. 1).

1 $${{\rm{Se}}}_{{\rm{recovery}}}\,=\,\frac{({{\rm{Se}}}_{{\rm{shoots}}}-{{\rm{Se}}}_{{\rm{ctrl}},{\rm{shoots}}})+({{\rm{Se}}}_{{\rm{grain}}}-{{\rm{Se}}}_{{\rm{ctrl}},{\rm{grain}}})\times 100}{{{\rm{Se}}}_{{\rm{applied}}}}$$

where Se_shoots_ and Se_grain_ are the amounts of Se (µg pot^−1^) measured in the shoots and grains respectively (as calculated from the dry weight and tissue Se concentration) and Se_ctrl,shoots_ and Se_ctrl,grain_ are the Se amounts in shoots and grain of the control plants.

### Statistical analyses

The effects of different fertilization treatments on grain yield and Se concentrations were determined using the analysis of variance (ANOVA) procedure in SPSS (IBM SPSS Statistics for Windows, Version 24.0., IBM Corp, Armonk, New York), with a significance threshold of 5%. Duncan’s and Tukey’s post-hoc tests were used to compare treatment means.

## Supplementary information

Supplementary Information
