# Author Correction: Improving the efficacy of selenium fertilizers for wheat biofortification

**DOI:** 10.1038/s41598-020-74207-5

**Published:** 2020-10-07

**Authors:** Chandnee Ramkissoon, Fien Degryse, Rodrigo C. da Silva, Roslyn Baird, Scott D. Young, Elizabeth H. Bailey, Mike J. McLaughlin

**Affiliations:** 1 Fertiliser Technology Research Centre, School of Agriculture, Food and Wine, Waite Campus, University of Adelaide, Adelaide, South Australia Australia; 2 School of Biosciences, University of Nottingham, Sutton Bonington Campus, Loughborough, UK

Correction to: *Scientific Reports* 10.1038/s41598-019-55914-0, published online 20 December 2019

This Article contains a typographical error in the Methods section under subheading ‘Selenium fertilizers’ where,

‘For the treatment with the soil-applied selenate only, a Na2SeO4 solution containing 0.042 mg Se L − 1 was applied to the soil as 3 × 26 µL droplets, in the same position as the granular fertilizers.’

should read:

‘For the treatment with the soil-applied selenate only, a Na2SeO4 solution containing 0.042 g Se L − 1 was applied to the soil as 3 × 26 µL droplets, in the same position as the granular fertilizers.’

