# Supplementary material for: Improving the efficacy of selenium fertilizers for wheat biofortification
Source: Sci Rep. 2019 Dec 20;9:19520. doi: 10.1038/s41598-019-55914-0 (PMC6925308; doi:10.1038/s41598-019-55914-0)
Supplement: Supplementary file 1 — Supplementary Information [file 41598_2019_55914_MOESM1_ESM.pdf]

Supplementary information

Improving the efficacy of selenium fertilizers for wheat biofortification

Chandnee Ramkissoon[a,b*], Fien Degryse[a], Rodrigo C. da Silva[a], Roslyn Baird[a], Scott D. Young[b], Elizabeth H. Bailey[b] and Mike J. McLaughlin[a]

[a]School of Agriculture, Waite Campus, University of Adelaide, Glen Osmond, South Australia; [b]School of Biosciences, University of Nottingham, Sutton Bonington Campus, Loughborough, UK

*Corresponding author

Email: chandnee.ramkissoon@adelaide.edu.au

stxcr5@nottingham.ac.uk

Orcid ID: https://orcid.org/0000-0002-3395-8835

Table S1: HPLC-ICPMS operating conditions for Se speciation of grain samples

Isotopes monitored: ^{76}Se, ^{77}Se, ^{78}Se and ^{82}Se
Analytical column: Hamilton PRP-X100 anion exchange column (Phenomenex) (250 x 4.6 mm, 10 µm)
Column temperature: 25°C
Mobile phase: 10 mM citric acid with 2 % methanol (v/v); pH= 5
Flow rate: 0.8 mL min^{-1}
Injection volume: 100 µL
Tune conditions: H_2 reaction gas

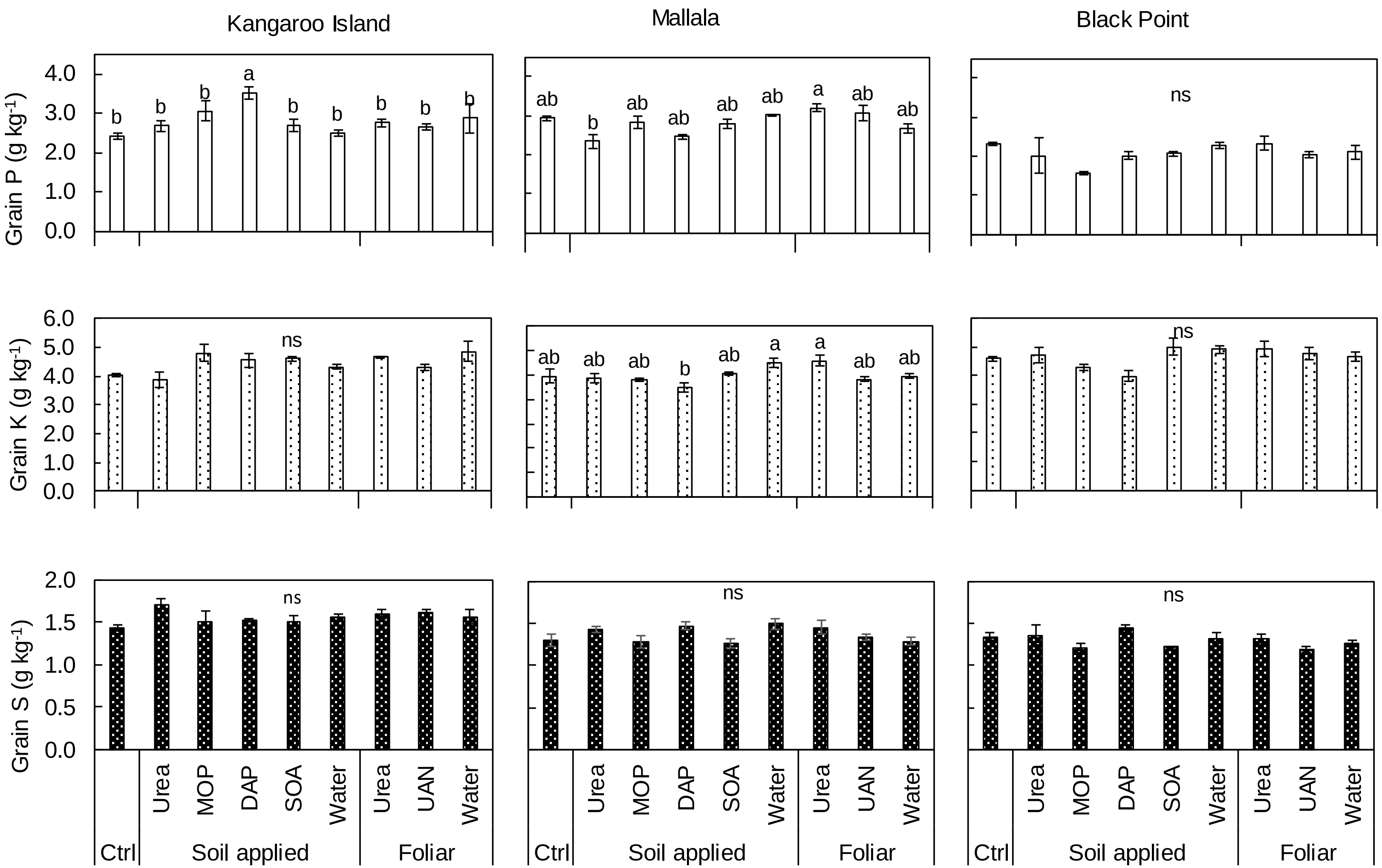

Fig. S1: Average concentrations of P, K and S in wheat grains grown under different fertilizer treatments (soil-applied and foliar) in three soils. Under soil-applied treatments, fertilizers urea, MOP, DAP and SOA supplying the macronutrients N, K, P and S respectively, were applied in granular form to the soil directly. In all other treatments, macronutrients were applied at the same rate in liquid form as a basal solution mixed

into the soil prior to potting. Control treatment (no Se applied) is denoted as ‘Ctrl’. The error bars represent standard errors (n=4); different letters represent significant differences in macronutrient concentration of grains under the different treatments, and ns denotes no statistical differences (Tukey’s test at 5% significance level).

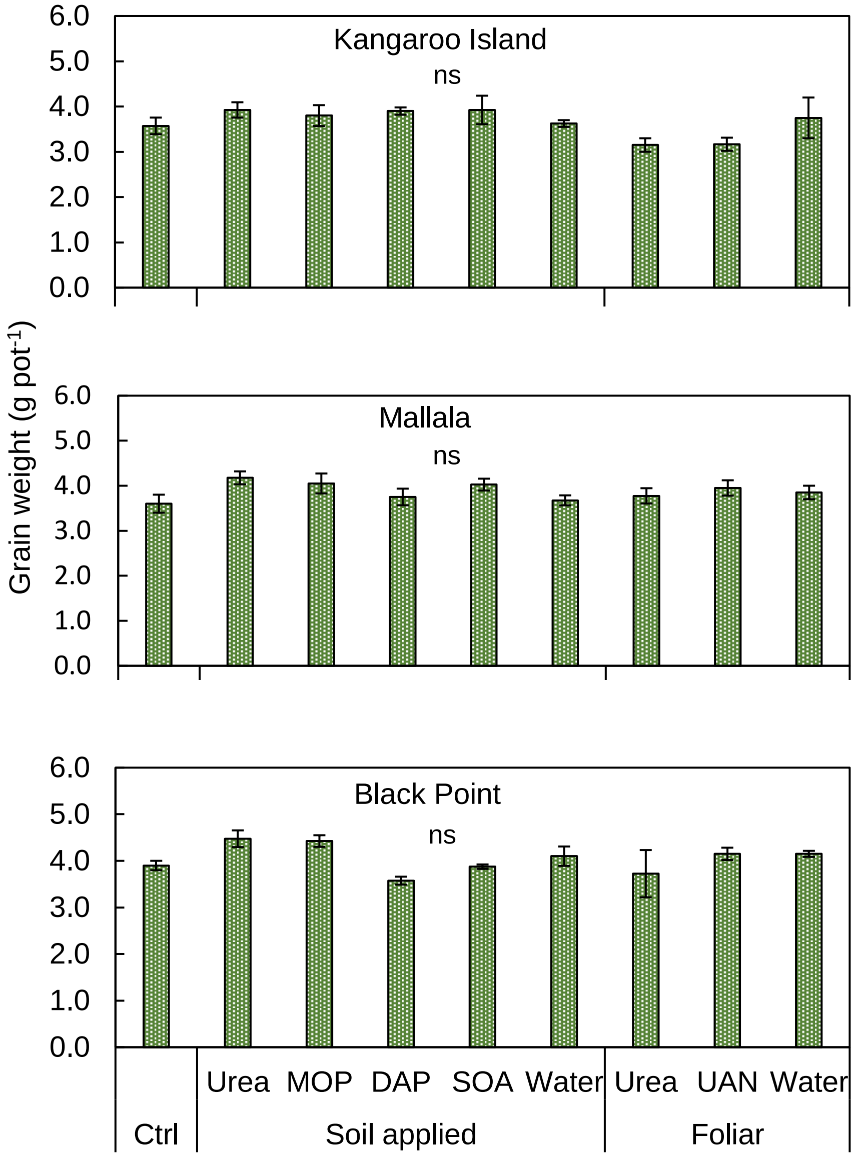

Fig. S2: Grain yield, measured as the dry weight of wheat grains per pot, across the different treatments for plants grown in three soils. The error bars represent standard errors (n=4) and ns denotes no statistical differences observed in grain yield across the different Se fertilizer treatments at a 5% significance level.

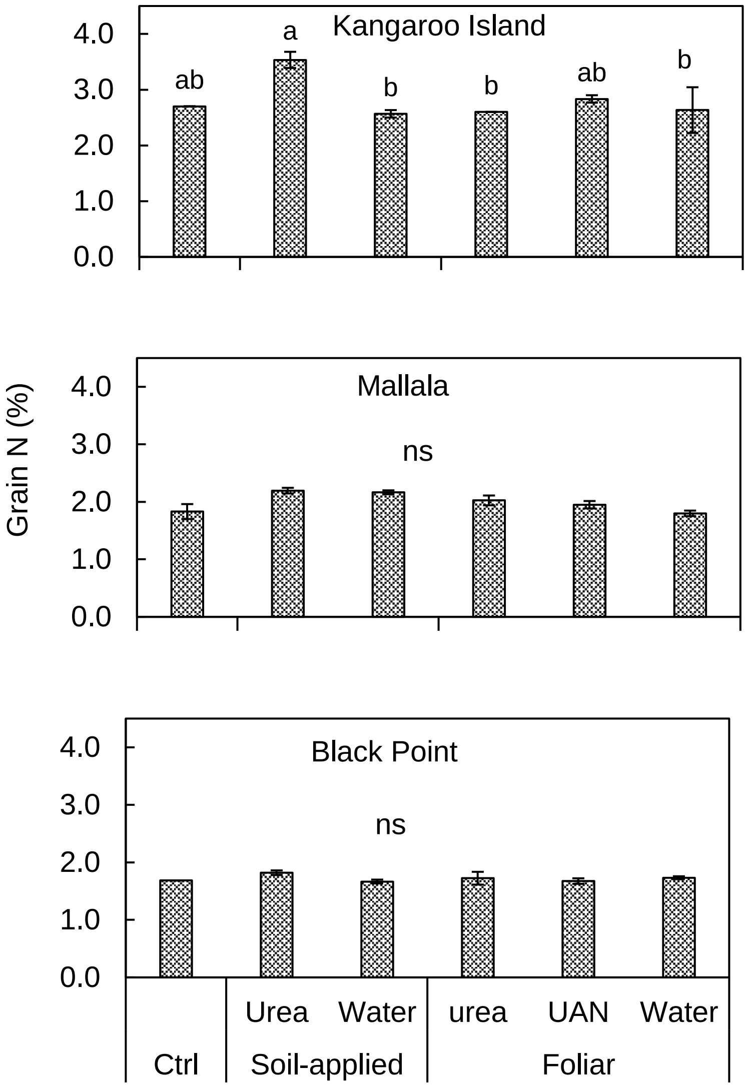

Fig. S3: Nitrogen content of grains of plants that were treated with Se-enriched N fertilizers as well as Se on its own (water as carrier) either to the soil or to the leaves, and grown in three different soils. The error bars show standard errors (n=4) and a,b represent Tukey's statistical differences at 5% significance level. ns denotes no significant differences in grain N content across the different fertilizer treatments.

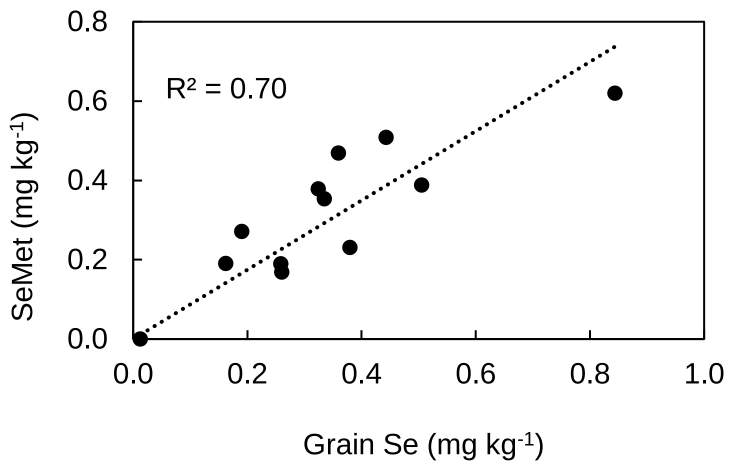

Fig. S4: Correlation between total Se and selenomethionine concentration of grains for foliar and soil-applied Se treatments
